# Kaiso Is a Novel TAZ-Interaction Partner That Promotes Hepatic Stellate Cell Activation and Regulates the Downstream lncRNA HIF1A-AS3

**DOI:** 10.3390/ijms27177847

**Published:** 2026-09-02

**Authors:** Amira Khaled Abdelhamid, Fabiola Pedrini, Jennifer Schmitt, Ibrahim Hassan Fayed, Aya Osama, Hannah H. Rashwan, Diana M.F. Hanna, Esther T. Menze, Sameh Magdeldin, Marcin Luzarowski, Carsten Sticht, Ebtehal El-Demerdash, Kai Breuhahn, Ahmed Ihab Abdelaziz, Nada El-Ekiaby

**Affiliations:** 1 School of Medicine, Newgiza University (NGU), Giza 12577, Egypt; amira.abdelhamid@ngu.edu.eg (A.K.A.); ibrahim.hassan@ngu.edu.eg (I.H.F.); aihab@ngu.edu.eg (A.I.A.); 2 Department of Pharmacology and Toxicology, Faculty of Pharmacy, Ain Shams University, Cairo 11566, Egypt; diana_magdy_fahim@pharma.asu.edu.eg (D.M.F.H.); esther.tharwat@pharma.asu.edu.eg (E.T.M.); ebtehal_dm@yahoo.com (E.E.-D.); 3 Institute of Pathology, University Hospital Heidelberg, 69120 Heidelberg, Germany; fabiola.pedrini@med.uni-heidelberg.de (F.P.); jennifer.schmitt@med.uni-heidelberg.de (J.S.); kai.breuhahn@med.uni-heidelberg.de (K.B.); 4 Proteomics and Metabolomics Research Program, Research Department, Children’s Cancer Hospital Egypt 57357 (CCHE-57357), Cairo 11562, Egypt; aya_osama12@hotmail.com (A.O.); hannah.hisham@57357.org (H.H.R.); sameh.magdeldin@57357.org (S.M.); 5 Department of Physiology, Faculty of Veterinary Medicine, Suez Canal University, Ismailia 41522, Egypt; 6 Core Facility for Mass Spectrometry and Proteomics, Center for Molecular Biology, Heidelberg University (ZMBH), 69120 Heidelberg, Germany; m.luzarowski@zmbh.uni-heidelberg.de; 7 Core Facility Next Generation Sequencing, Medical Faculty Mannheim, Heidelberg University, 68167 Mannheim, Germany; carsten.sticht@medma.uni-heidelberg.de; 8 Preclinical and Translational Research Center, Faculty of Pharmacy, Ain Shams University, Cairo 11566, Egypt

**Keywords:** liver fibrosis, hepatic stellate cells, Hippo signaling pathway, yes-associated protein, transcriptional coactivator with PDZ-binding motif, long non-coding RNA, Kaiso, HIF1A-AS3

## Abstract

Yes-associated protein (YAP) and transcriptional coactivator with PDZ-binding motif (TAZ) are key regulators of hepatic stellate cell (HSC) activation and liver fibrosis. While the canonical YAP/TAZ-TEAD axis is well-characterized, non-canonical interaction partners and downstream long non-coding RNAs (lncRNAs) that orchestrate fibrogenic programs in HSCs remain undefined. Using BioID and next-generation sequencing (NGS) in LX-2 cells, we identified YAP/TAZ-interactomes and YAP/TAZ-regulated lncRNAs. BioID results and proximity ligation assay (PLA) identified the transcription factor Kaiso as a TAZ interaction partner. Functionally, Kaiso knockdown suppressed key aspects of HSC activation in LX-2 cells. We next integrated our NGS results from YAP/TAZ-knockdown in LX-2 cells with publicly available Kaiso ChIP-seq data. This identified HIF1A-AS3 as a common downstream lncRNA. ChIP-qPCR confirmed direct binding of Kaiso to the *HIF1A-AS3* promoter. Furthermore, HIF1A-AS3 silencing mirrored the functional effects of Kaiso knockdown. Global proteomic profiling following knockdown of TAZ, Kaiso, or HIF1A-AS3 revealed a network of commonly regulated proteins enriched for pathways central to HSC activation. In conclusion, we establish Kaiso as a novel TAZ interaction partner and identify HIF1A-AS3 as a shared downstream target of TAZ and Kaiso, suggesting a potential profibrotic regulatory network that promotes HSC activation and may represent a therapeutic target for liver fibrosis.

## 1. Introduction

Liver fibrosis arises from the continuous inflammation and wound-healing responses associated with chronic liver diseases [1,2,3]. This pathological process results in the excessive deposition of extracellular matrix (ECM) within the liver, leading to progressive scarring and disruption of hepatic architecture and function [4,5]. If left untreated, liver fibrosis can further advance to cirrhosis, liver failure, or hepatocellular carcinoma (HCC) [3]. A key event in the development of liver fibrosis is the activation of hepatic stellate cells (HSCs) [6,7], which, in response to liver injury, transdifferentiate into fibrogenic, ECM-producing myofibroblasts [8]. Multiple signaling pathways drive the activation of HSCs, including the TGF-β pathway [9], the Wnt/β-catenin pathway [10], and the recently implicated Hippo pathway [11,12].

The Hippo pathway, originally recognized for its role in regulating organ size and maintaining tissue homeostasis, plays a central role in liver fibrosis [13,14,15]. Normally, an active Hippo pathway results in cytoplasmic retention and proteasomal degradation of the transcriptional co-activators yes-associated protein (YAP) and transcriptional co-activator with PDZ-binding motif (TAZ) [16,17,18]. As a result, YAP/TAZ-driven target gene transcription is repressed [16]. On the other hand, upon liver injury, the Hippo pathway is suppressed, resulting in YAP/TAZ dephosphorylation and nuclear translocation, where they activate genes crucial for tissue regeneration [12]. However, sustained nuclear activity of YAP/TAZ promotes excessive HSC activation and induces key fibrogenic genes, such as alpha-smooth muscle actin *(α-SMA),* thereby contributing to liver fibrosis [14,15,19,20]. Since YAP/TAZ lack intrinsic DNA-binding capability, their ability to modulate transcription and regulate fibrogenic genes relies heavily on interactions with other transcription factors and cofactors [21].

The most well-characterized interaction partners of YAP/TAZ are the TEA domain (TEAD) family of transcription factors, and in liver fibrosis, the YAP/TAZ-TEAD axis has been implicated in driving the transcription of fibrogenic genes, such as *CD47*, and promoting HSC activation [21,22,23,24]. Interestingly, while this axis has received the most attention in liver fibrosis and across diverse physiological and pathological contexts, YAP/TAZ have also been shown to interact with a wider range of transcription factors. These interactions critically influence their functional outcomes [21]. For example, YAP/TAZ play an oncogenic role by interacting with STAT3 in breast cancer, driving *IL6* transcription and promoting inflammation and oncogenic transformation [25]. In contrast, YAP acts as a tumor suppressor in lung cancer by interacting with p73 and promoting transcription of the pro-apoptotic gene *Bax* [26]. These observations highlight the importance of YAP/TAZ interaction partners in shaping their transcriptional output and downstream biological effects. Nevertheless, the influence of such non-canonical partners on YAP/TAZ target gene regulation, including long non-coding RNAs (lncRNAs), in HSCs remains largely unexplored.

lncRNAs are increasingly recognized for their role in regulating gene expression and cellular functions [27,28,29]. In liver fibrosis, they have been shown to play critical roles in HSC activation. For instance, lnc-LFAR1, PVT1, and SNHG5 were found to impact crucial fibrogenic pathways, including the TGF-β, Notch, and Hippo pathways [30,31,32]. Interestingly, YAP/TAZ have been reported to regulate lncRNAs in various biological contexts [33,34], and our group recently characterized a YAP/TAZ-regulated lncRNA signature in HCC [35]. On the other hand, RNA sequencing of differentially expressed lncRNAs in activated HSCs revealed that the Hippo pathway is one of the most significantly enriched pathways associated with these lncRNAs [36]. Despite the well-established fibrogenic roles of YAP/TAZ and the recognized potential of lncRNAs to mediate these effects, the regulation of lncRNAs by YAP/TAZ and their interaction partners in HSCs, and their contribution to fibrogenic activity, remains poorly defined.

To address this gap, we employed BioID proximity labeling to identify novel YAP/TAZ interaction partners and RNA sequencing to define downstream YAP/TAZ-regulated lncRNAs in HSCs. The experiments were conducted using LX-2 cells, a well-established immortalized human HSC model widely used to investigate HSC activation and fibrogenic mechanisms [37,38]. Our work uncovered new fibrogenic mediators, identifying Kaiso as a novel TAZ interaction partner and HIF1A-AS3 as a downstream lncRNA.

## 2. Results

### 2.1. BioID Identifies Common and Unique Interaction Partners for YAP and TAZ in HSCs

To identify YAP/TAZ interaction partners, we mapped their interactomes in the LX-2 cell line using BioID coupled with LC–MS/MS. For this purpose, we generated stable LX-2 cells expressing doxycycline-inducible BirA-Flag-YAP or BirA-Flag-TAZ fusion proteins to enable proximity-dependent biotinylation. Following doxycycline treatment, successful induction of the BirA-Flag-YAP and BirA-Flag-TAZ constructs was confirmed by immunoblot analysis, which demonstrated the expected molecular-size shifts for YAP and TAZ and detected the Flag tag at the anticipated sizes (Supplementary Figure S1A). To validate the functionality of the BirA-YAP and BirA-TAZ fusion proteins, we performed streptavidin blotting. Cells treated with doxycycline and biotin exhibited robust biotinylation of proteins proximal to YAP and TAZ, as indicated by the characteristic laddering pattern, a hallmark of successful BioID (Supplementary Figure S1B). These results demonstrate that the BirA-YAP and BirA-TAZ fusion proteins were functional and suitable for subsequent proteomic profiling.

**Figure 1:**
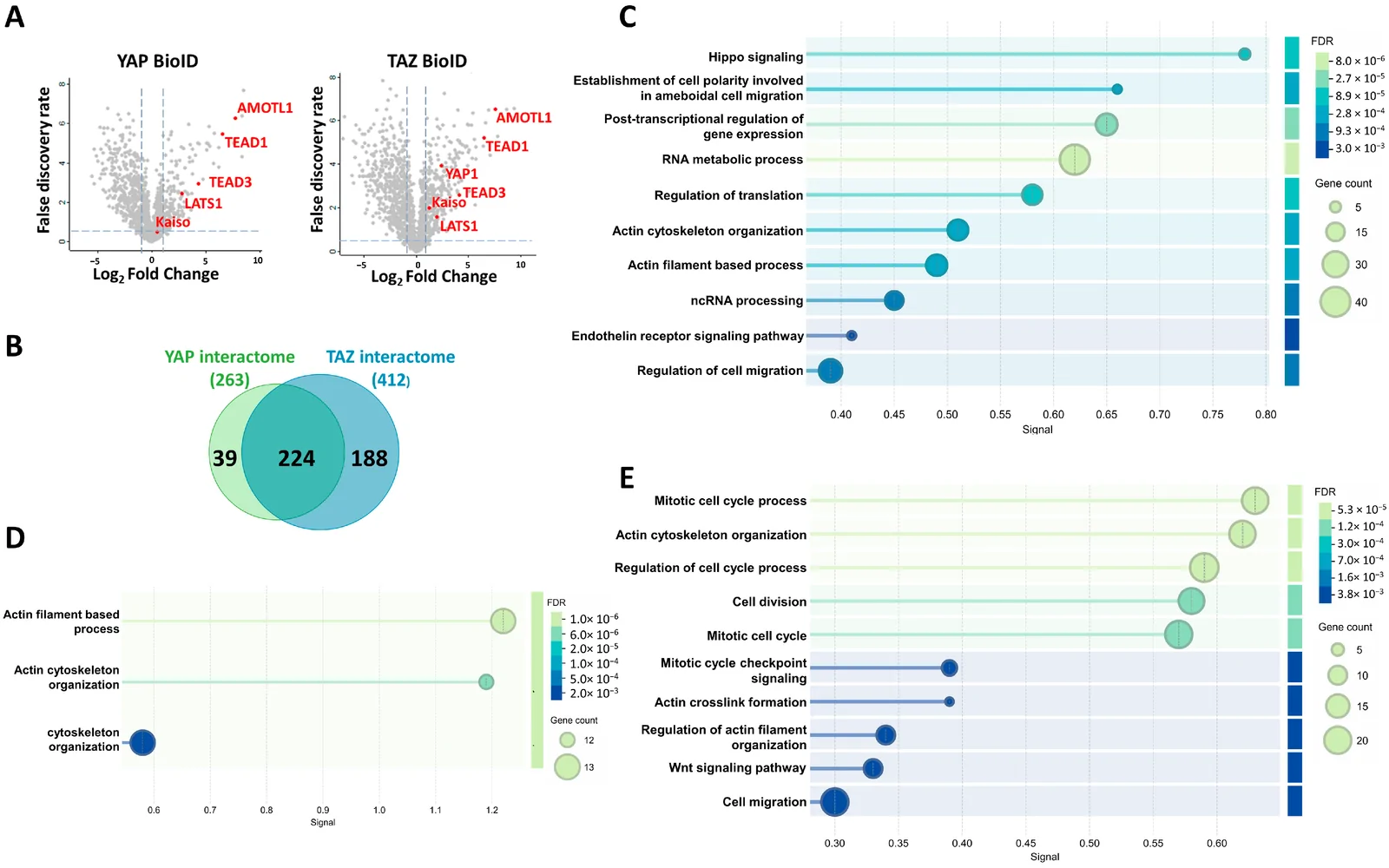
BioID Mapping and GO-BP Enrichment Analysis of YAP and TAZ Interactomes in LX-2 Cells. BioID followed by LC–MS/MS analysis was performed to map the YAP and TAZ interactomes in LX-2 cells, and GO-BP enrichment analysis was conducted using the STRING database. (**A**) Volcano plots showing proteins significantly enriched in the YAP (left) and TAZ (right) interactomes compared with the empty vector control. (**B**) Venn diagram illustrating the overlap between proteins identified in the YAP and TAZ interactomes. (**C**) GO-BP enrichment analysis of proteins shared between the YAP and TAZ interactomes; representative terms are shown to reduce redundancy. (**D**) GO-BP enrichment analysis of proteins unique to the YAP interactome. (**E**) GO-BP enrichment analysis of proteins unique to the TAZ interactome; representative GO terms are shown to reduce redundancy.

LC/MS analysis of the biotinylated fraction was performed, revealing a diverse network of proteins interacting with YAP and TAZ. Only proteins significantly enriched compared to the BirA-only control (fold change ≥ 2; false discovery rate ≤ 0.05) were considered putative YAP/TAZ interaction partners. Established interactors such as TEAD transcription factors, angiomotins (AMOTL1/2), and the kinase LATS1 were detected, confirming the robustness of our BioID approach. In addition to these known partners, we also identified previously unrecognized interactors of YAP and TAZ. In total, 263 interactors were characterized for YAP and 412 for TAZ, represented as volcano plots (Figure 1A). A comparison of the two interactomes showed that 224 interaction partners are shared, whereas 39 are unique to YAP and a larger set of 188 are unique to TAZ, as visualized in a Venn diagram (Figure 1B).

### 2.2. Enrichment Analysis Reveals YAP/TAZ Interactomes Are Associated with Key Processes in HSCs

Given that our BioID results revealed both common and exclusive interaction partners for YAP and TAZ, we performed Gene Ontology (GO) enrichment analysis using the STRING database to explore the potential functional roles of each interactome. The common YAP/TAZ interactome (224 interactors) showed robust enrichment for a wide range of biological processes. As expected, the Hippo signaling pathway was among the most significantly enriched, together with processes consistent with their canonical role in mechanotransduction. Beyond these known functions, the analysis uncovered a significant role for YAP/TAZ in controlling RNA fate and protein synthesis (Figure 1C, Supplementary Table S4). The YAP-specific interactome (39 interactors) revealed only three significantly enriched terms, all associated with cytoskeletal organization (Figure 1D, Supplementary Table S5). In contrast, the TAZ-exclusive interactome (188 interactors) displayed a larger network with 179 enriched terms. The most dominant functional theme reflected a role in cell proliferation. A second major theme, similar to the YAP interactome, involved the regulation of the cytoskeleton organization. Furthermore, other terms highly relevant to HSC activation were also significantly enriched, including cell migration and Wnt signaling (Figure 1E, Supplementary Table S6).

Collectively, these findings demonstrate that YAP and TAZ share a core set of interaction partners that, aside from their canonical role, have a function in shaping gene expression via post-transcriptional and translational mechanisms. Additionally, each also possesses a distinct subset of exclusive interactors. Thus, despite their overlapping functions as paralogs, YAP and TAZ may fulfill specialized roles in HSCs, with YAP preferentially associated with cytoskeletal organization and TAZ exhibiting a more diverse role encompassing proliferation, cytoskeletal organization, migration, and other parameters of HSC activation.

### 2.3. The Transcription Factor Kaiso Is a TAZ Interaction Partner

**Figure 2:**
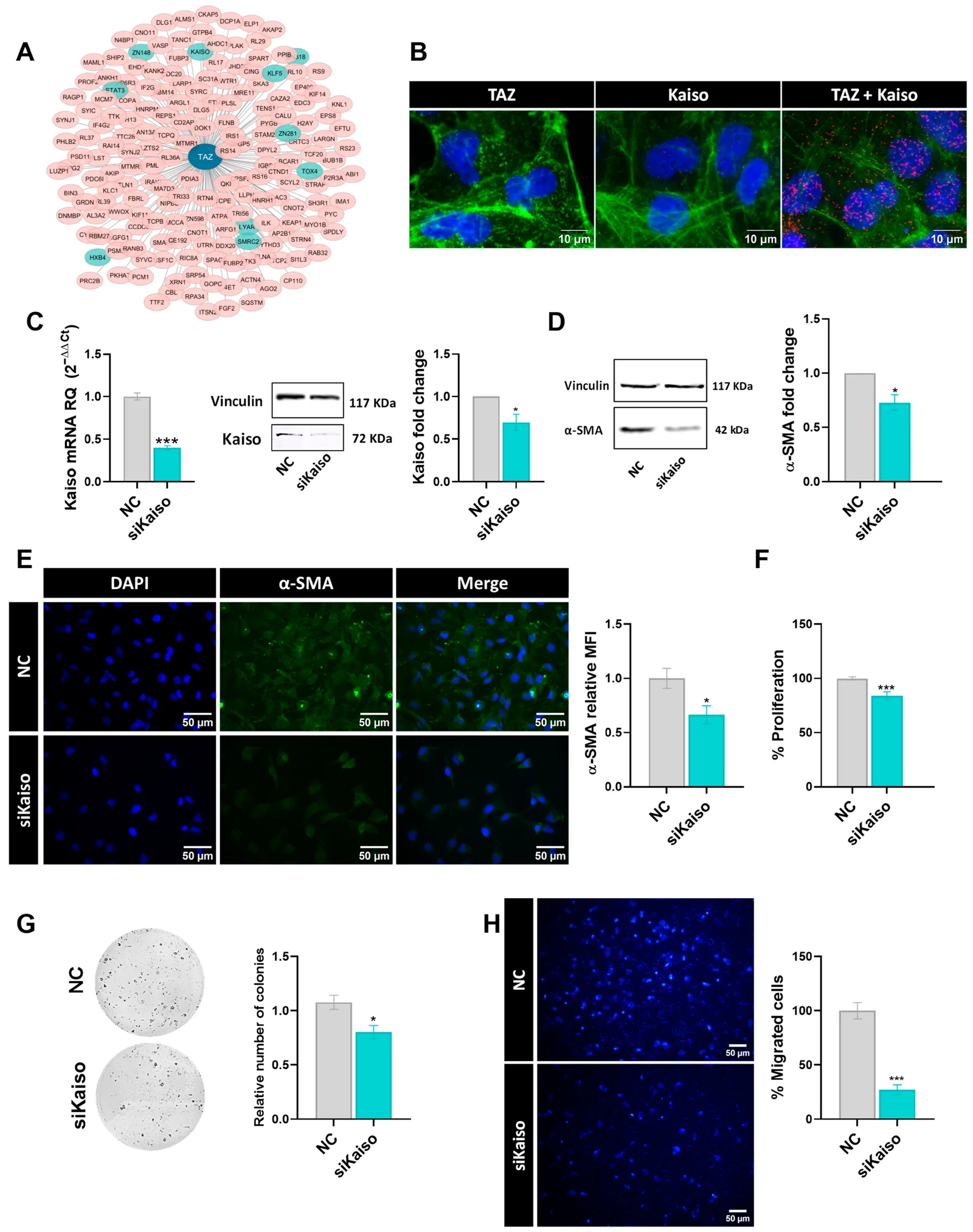
Identification of Kaiso as a TAZ interaction partner and its functional characterization in hepatic stellate cells (HSCs). (**A**) Cytoscape network depicting TAZ-exclusive interaction partners identified by BioID. Predicted transcription factors are highlighted in turquoise. (**B**) Validation of the TAZ-Kaiso interaction by proximity ligation assay (PLA). Left, anti-TAZ antibody only; middle, anti-Kaiso antibody only; right, anti-TAZ and anti-Kaiso antibodies. Red dots indicate PLA signals, green indicates phalloidin (F-actin), and blue indicates DAPI (nuclei). (**C**) Validation of Kaiso knockdown efficiency in LX-2 cells transfected with Kaiso-targeting siRNAs (siKaiso) or a non-targeting negative control (NC) by qRT-PCR (left) and Western blot analysis with corresponding quantification (middle and right). Vinculin served as the loading control. (**D**) Western blot analysis (left) and quantification (right) of α-SMA protein levels in NC and siKaiso-transfected cells. Vinculin served as the loading control. (**E**) Immunofluorescence images of α-SMA (green) and nuclei (DAPI, blue) in NC and siKaiso-transfected cells (left) and quantification of mean fluorescence intensity (MFI) (right). (**F**) BrdU assay measuring proliferation in NC and siKaiso-transfected cells. (**G**) Representative images of colony formation assay (left) showing NC (top) and siKaiso-transfected cells (bottom), with quantification of relative colony numbers (right). (**H**) Representative images of Transwell migration assay (left) showing NC (top) and siKaiso-transfected cells (bottom), with quantification of the percentage of migrated cells (right). Statistical significance: * *p* < 0.05, *** *p* < 0.001.

Given the extensiveness of the TAZ interactome and the diversity of its enriched pathways, we focused our analysis on its interaction partners. We were particularly interested in those with transcriptional regulatory activity. To identify these candidates, proteins identified by BioID as interacting with TAZ only were filtered for transcription factors using the AnimalTFDB database (Figure 2A). From this list, we prioritized transcription factors that had not yet been investigated in liver fibrosis but showed evidence of regulating fibrotic pathways in other contexts. Based on this rationale, we selected the transcription factor Kaiso.

Kaiso is a bimodal transcription factor capable of both activating and repressing gene transcription in a context-dependent manner [39,40]. Since its discovery, Kaiso has been linked to several major signaling pathways, including Wnt/β-catenin, TGF-β, and Notch [40,41,42,43,44]. Although these pathways are known to be central to HSC activation, Kaiso’s role has only been studied in other contexts, such as pulmonary fibrosis, where it was found to exert pro-fibrotic effects [45]. In the liver, however, studies are limited, with one study reporting that Kaiso promotes cell-cycle progression and tumor growth in HCC [46], while another showed that it is overexpressed in metabolic dysfunction-associated steatotic liver disease (MASLD) patients and associated with the progression of MASLD to HCC [47]. Despite these findings, its specific contribution to liver fibrosis is yet to be elucidated.

To further explore the possible functional relevance of Kaiso in the context of HSCs, we utilized a recently published HSC atlas [48]. This resource integrates single-cell sequencing data from 10 mouse datasets across various liver injury models (*n* = 57,233 HSCs) and 3 human datasets (*n* = 774 HSCs), and identifies three HSC populations: quiescent HSCs, initiatory HSCs, and myofibroblasts. The authors next inferred transcription factor activity across these populations. Interestingly, this atlas revealed that in mice, Kaiso activity was higher in myofibroblasts compared to quiescent and initiatory HSCs (Supplementary Figure S2A). In humans, a similar trend was observed, though the difference was more subtle, likely attributable to the smaller sample size (Supplementary Figure S2B). Collectively, these findings identify Kaiso as a potential regulator of HSC activation.

Given that Kaiso was identified as a candidate TAZ interactor in our BioID screen, we next sought to validate this association experimentally using proximity ligation assay (PLA). A robust PLA signal was observed between endogenous TAZ and Kaiso, supporting their close spatial association in cells (Figure 2B).

### 2.4. Kaiso Regulates Fibrogenic Activation of HSCs

We next examined its role in regulating α-SMA. Notably, α-SMA is a reliable marker of HSC activation, reflecting their transition into a myofibroblast-like phenotype [49,50]. Under standard culture conditions, LX-2 cells exhibit features of an activated phenotype, including basal expression of α-SMA [37,38]. Previous work has shown that siRNA-mediated knockdown of TAZ reduces HSC activation and α-SMA expression in LX-2 cells [15]. Therefore, we investigated whether Kaiso knockdown could similarly affect α-SMA regulation. To this end, LX-2 cells were transfected with Kaiso-targeting siRNAs, resulting in a significant reduction in Kaiso mRNA and protein expression compared with the negative control (NC) (*p* < 0.0001 and *p* = 0.045, respectively) (Figure 2C). We then assessed the impact of Kaiso knockdown on α-SMA expression. Western blot analysis showed a significant decrease in α-SMA protein following Kaiso knockdown (*p* = 0.0204) (Figure 2D). Consistently, immunofluorescence staining demonstrated a marked reduction in α-SMA signal in Kaiso-knockdown cells relative to the negative control (*p* = 0.0215) (Figure 2E).

The effect of Kaiso knockdown was further investigated on functional parameters associated with HSC activation, including proliferation, migration, and colony formation. HSC proliferation was evaluated using the BrdU assay, which revealed a significant reduction in proliferation in Kaiso-knockdown cells (*p* = 0.0003) (Figure 2F). Colony formation assays demonstrated that Kaiso knockdown significantly impaired the ability of LX-2 cells to form colonies (*p* = 0.0302) (Figure 2G). Finally, to assess migration, a Transwell assay was performed, and Kaiso-silenced cells exhibited markedly reduced migratory capacity (*p* < 0.0001) (Figure 2H). Taken together, these results demonstrate that Kaiso is required for key HSC activation phenotypes including α-SMA expression, proliferation, colony formation, and migration, with the most pronounced reduction observed in migration assays.

### 2.5. Identification of HIF1A-AS3 as YAP/TAZ and Kaiso-Regulated lncRNA

**Figure 3:**
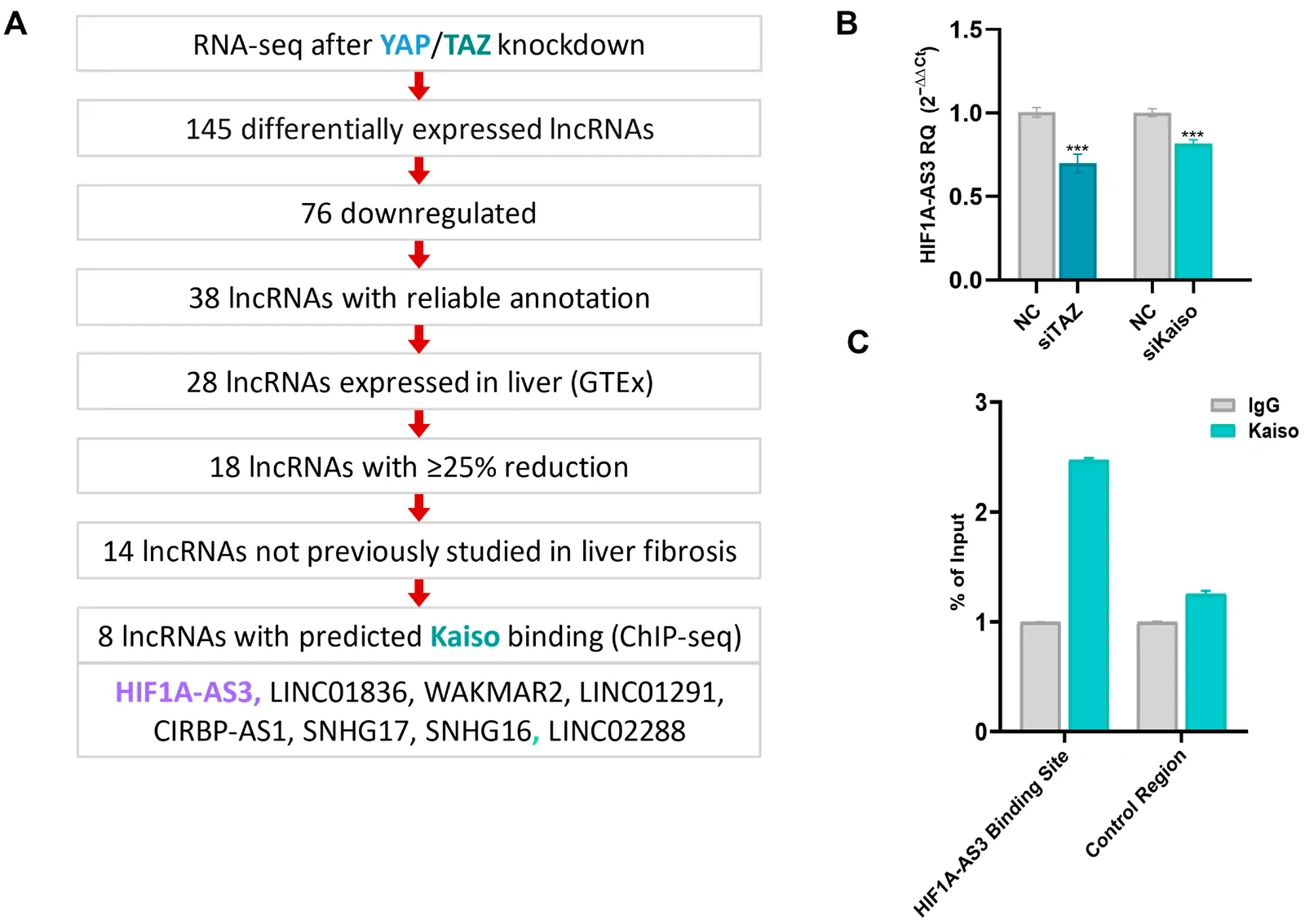
Identification and validation of HIF1A-AS3 as a YAP/TAZ- and Kaiso-regulated lncRNA. Candidate lncRNAs were identified through RNA sequencing of LX-2 cells following YAP/TAZ knockdown and integrated with publicly available Kaiso ChIP-seq data to prioritize lncRNAs with potential Kaiso binding. (**A**) Workflow diagram illustrating the filtering and selection strategy used to shortlist candidate lncRNAs. (**B**) qRT-PCR analysis of HIF1A-AS3 expression in LX-2 cells transfected with NC, siTAZ, or siKaiso. (**C**) ChIP-qPCR assay assessing Kaiso occupancy at the predicted *HIF1A-AS3* promoter, with IgG and a negative genomic region used as controls. Statistical significance: *** *p* < 0.001.

After comprehensively characterizing the non-canonical interaction landscape of YAP/TAZ in HSCs, which culminated in the identification of a novel interaction partner with functional relevance to HSC activation, we next investigated another underexplored regulatory layer of YAP/TAZ signaling, namely lncRNAs. We therefore sought to identify Hippo pathway-regulated lncRNAs that might mediate their profibrotic activity in HSCs and to investigate whether Kaiso intersects with this regulatory landscape. To avoid compensatory effects, both YAP and TAZ were simultaneously inhibited using gene-specific siRNAs, and efficient YAP/TAZ knockdown was confirmed using WB (Supplementary Figure S3A). Next, NGS following YAP/TAZ knockdown was performed, revealing 145 differentially expressed lncRNAs, including 76 that were downregulated and 69 that were upregulated. Given the established profibrotic role of YAP/TAZ signaling, we prioritized lncRNAs downregulated upon YAP/TAZ inhibition, as these are more likely to act as profibrotic mediators. We then further shortlisted the lncRNAs according to the following criteria: (I) reliable transcript annotation, (II) detectable liver expression according to the Genotype-Tissue Expression (GTEx) portal to ensure tissue relevance, (III) at least 25% downregulation upon YAP/TAZ knockdown to ensure biological significance, and (IV) no prior studies in liver fibrosis, as we aimed to characterize novel lncRNAs in this context. This filtering resulted in fourteen candidate lncRNAs (Figure 3A).

Considering our finding that Kaiso exerts a profibrotic role and given its establishment as a novel interaction partner within the Hippo pathway, we were intrigued to investigate the role of Kaiso in transcriptionally regulating lncRNAs downstream of YAP/TAZ. To explore this possibility, we further narrowed down the candidate lncRNAs using publicly available Kaiso ChIP-seq data, which identified eight lncRNAs with potential Kaiso-binding sites in the promoter (Figure 3A). Among these, we selected hypoxia-inducible factor 1 alpha antisense RNA 3 (HIF1A-AS3), as it showed the strongest downregulation following YAP/TAZ knockdown, implying robust Hippo pathway regulation. This decrease was confirmed by qRT-PCR (*p* < 0.0001) (Supplementary Figure S3B).

To validate the regulation of HIF1A-AS3 by TAZ and Kaiso, we assessed its levels following their knockdown. qRT-PCR revealed that HIF1A-AS3 expression was significantly reduced in cells silenced for TAZ (*p* < 0.0001) and Kaiso (*p* < 0.0001) (Figure 3B). To determine whether Kaiso directly regulates HIF1A-AS3 transcription, we performed ChIP analysis using primers targeting the predicted Kaiso-binding region in the *HIF1A-AS3* promoter. Percent input analysis showed clear enrichment of this region in samples immunoprecipitated with the Kaiso antibody compared with the IgG control, whereas a negative control region showed minimal signal, confirming specific binding of Kaiso at *HIF1A-AS3* promoter (Figure 3C).

### 2.6. HIF1A-AS3 Regulates Fibrogenic Activation of HSCs

**Figure 4:**
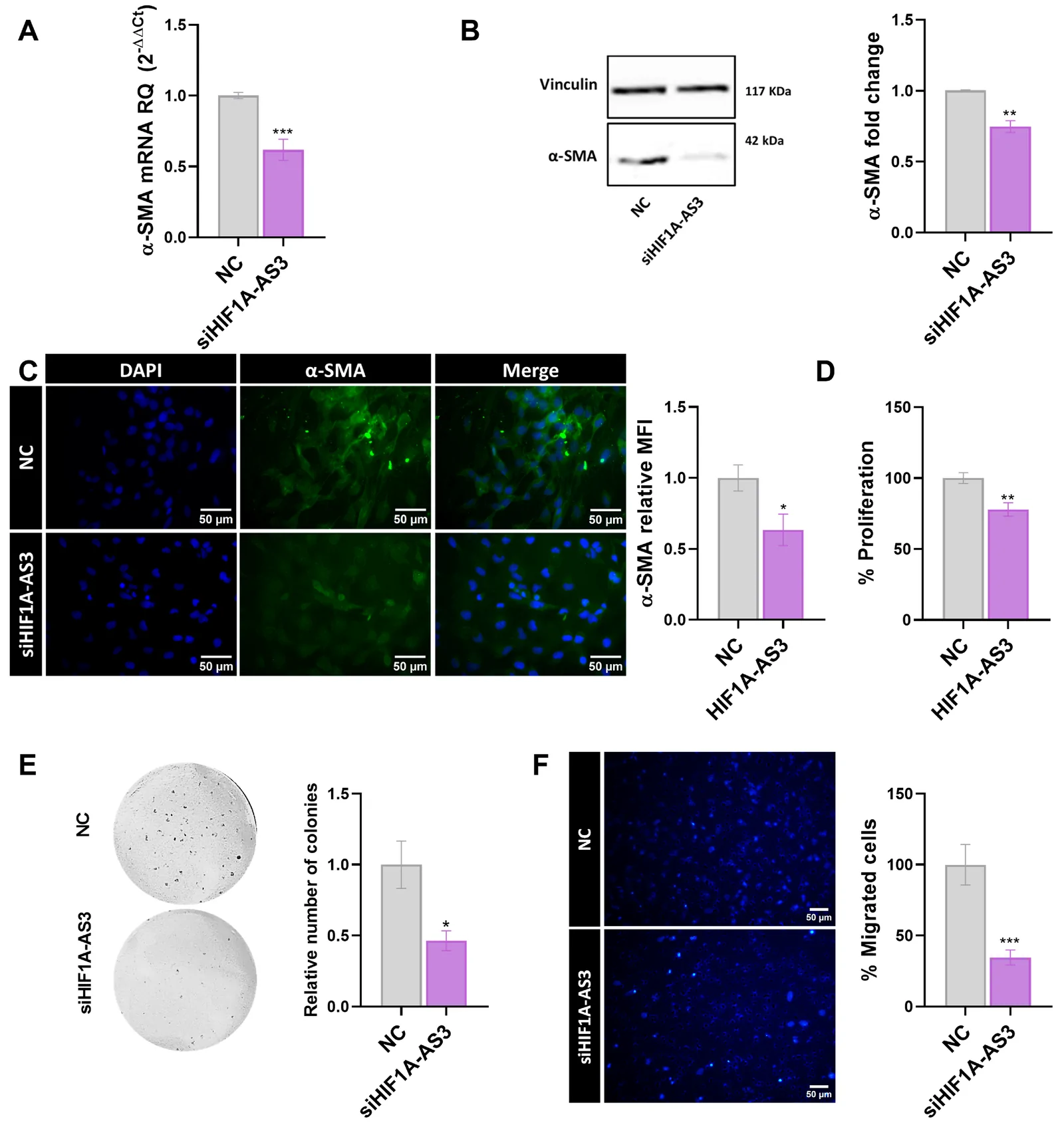
Impact of HIF1A-AS3 knockdown on HSC activation. HIF1A-AS3 KD was performed in LX-2 cells using siHIF1A-AS3, with non-targeting NC oligos used as the control. The effects of HIF1A-AS3 silencing on HSC activation marker expression and cellular functional behaviors were assessed. (**A**) qRT-PCR quantification of α-SMA mRNA levels in NC and siHIF1A-AS3–transfected cells. (**B**) Western blot analysis of α-SMA protein levels (left) and quantification (right) in NC and siHIF1A-AS3–transfected cells; vinculin served as a loading control. (**C**) Immunofluorescence images showing α-SMA (green) and nuclei (DAPI, blue) in NC and siHIF1A-AS3–transfected cells (left), with quantification of MFI (right). (**D**) BrdU assay measuring cell proliferation in NC and siHIF1A-AS3–transfected cells. (**E**) Representative images of colony formation assay (left) and relative quantification of colony numbers (right) in NC and siHIF1A-AS3–transfected cells. (**F**) Representative images of Transwell migration assay (left) and quantification of the percentage of migrated cells (right) in NC and siHIF1A-AS3–transfected cells. Statistical significance: * *p* < 0.05, ** *p* < 0.01, *** *p* < 0.001.

Because HIF1A-AS3 was identified as a lncRNA regulated downstream of YAP/TAZ and as a direct transcriptional target of Kaiso, we next examined its functional role in HSC activation. siRNA-mediated knockdown of HIF1A-AS3 was performed, and efficient silencing was confirmed by qRT-PCR, which demonstrated a significant reduction in HIF1A-AS3 levels (*p* = 0.0006) (Supplementary Figure S3C). This reduction was accompanied by a significant decrease in α-SMA mRNA expression (*p* = 0.0001) (Figure 4A). Consistently, Western blot analysis showed a significant decrease in α-SMA protein levels in knockdown cells (*p* = 0.0037) (Figure 4B). Immunofluorescence staining further confirmed a significant reduction in α-SMA signal intensity in HIF1A-AS3-silenced cells (*p* = 0.0284) (Figure 4C). The BrdU proliferation assay showed that HIF1A-AS3 silencing significantly reduced cell proliferation (*p* = 0.0021) (Figure 4D). Colony formation assays demonstrated a significant impairment in clonogenic capacity following knockdown (*p* = 0.0418) (Figure 4E). Additionally, Transwell migration assays revealed a significant decrease in the number of migrated cells upon HIF1A-AS3 silencing (*p* = 0.0005) (Figure 4F). Together, these results indicate that HIF1A-AS3 contributes to HSC activation.

### 2.7. TAZ, Kaiso, and HIF1A-AS3 Regulate Overlapping Proteomic Networks in HSCs

**Figure 5:**
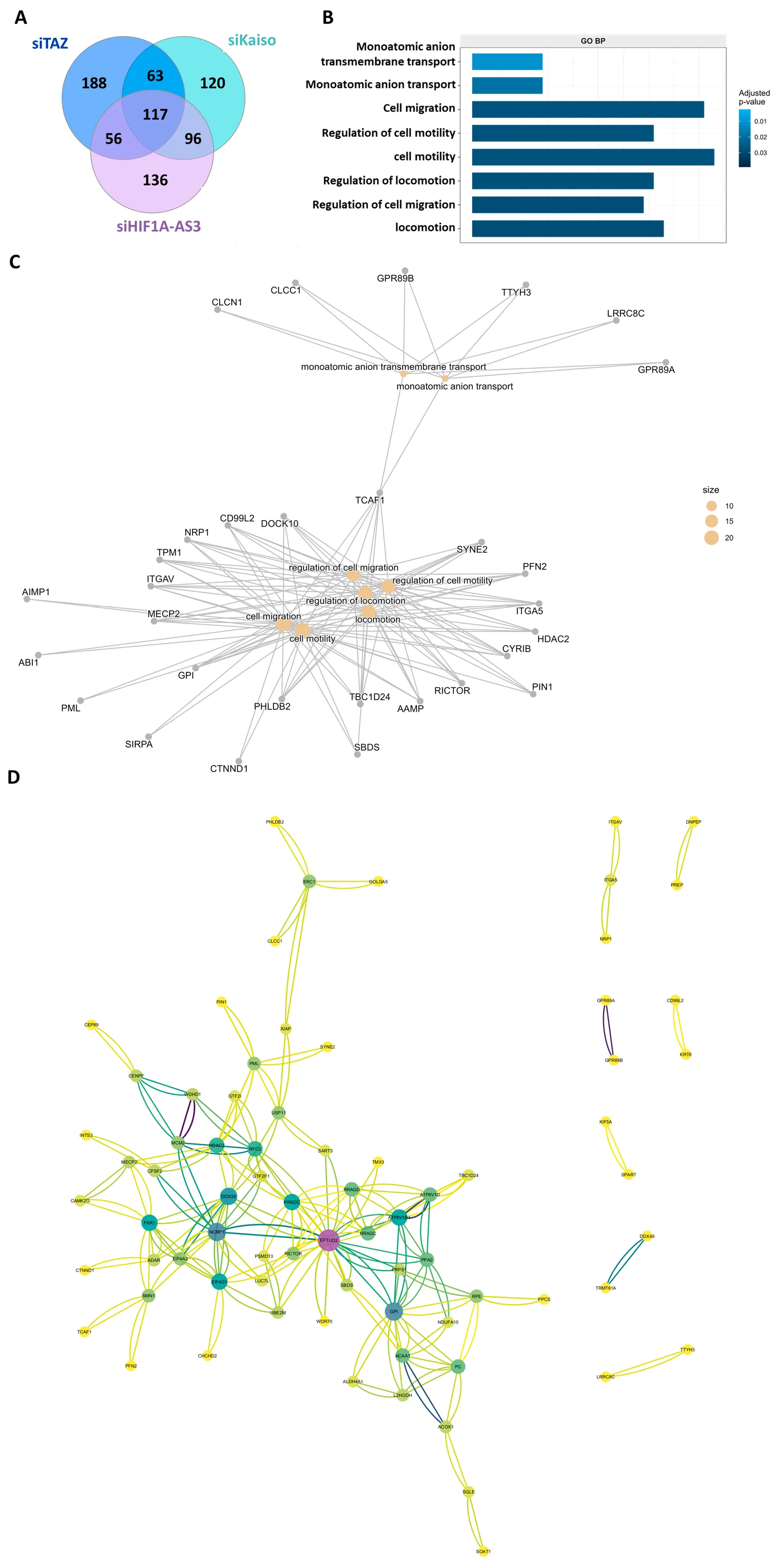
Whole-proteome analysis following TAZ, Kaiso, and HIF1A-AS3 knockdown in LX-2 cells. LC-MS/MS proteomic analysis was performed following TAZ, Kaiso, or HIF1A-AS3 knockdown to identify differentially expressed proteins (DEPs). (**A**) Venn diagram showing the overlap of DEPs across the three knockdown conditions. (**B**) Bar plot showing significantly enriched GO-BP terms for the shared DEPs. Bars represent enrichment magnitude, while color intensity indicates the adjusted *p*-value following Benjamini–Hochberg correction. (**C**) Enrichment network analysis of the shared DEPs illustrating significantly enriched functional terms and their associated proteins. (**D**) A protein–protein interaction (PPI) network constructed using STRING for the overlap gene set. Nodes represent proteins, while edges indicate known or predicted interactions. Node size and color intensity correspond to degree centrality. Highly connected nodes (hub genes) are highlighted. Edge colors denote interaction evidence derived from co-expression and experimentally determined data, and edge thickness reflects interaction confidence.

To investigate whether TAZ, Kaiso, and HIF1A-AS3 exert convergent regulatory effects in HSCs, we performed whole-proteome analysis to identify differentially expressed proteins (DEPs) following knockdown of TAZ, Kaiso, or HIF1A-AS3. DEPs were defined as those with an adjusted *p*-value < 0.05 and an absolute log_2_ fold change ≥ 1. This approach identified 425 DEPs for TAZ, 397 for Kaiso, and 406 for HIF1A-AS3. Comparative analysis showed that out of all the DEPs, 117 proteins were commonly altered across all three knockdowns, as depicted in the Venn diagram (Figure 5A, Supplementary Table S7). Pairwise Spearman correlation analysis on these 117 common DEPs revealed strong positive correlations between the treatments (TAZ–Kaiso: r = 0.8127, *p* < 0.0001; TAZ–HIF1A-AS3: r = 0.8052, *p* < 0.0001; Kaiso–HIF1A-AS3: r = 0.8555, *p* < 0.0001).

To assess the functional relevance of the 117 common DEPs identified across the TAZ, Kaiso, and HIF1A-AS3 knockdowns, we performed GO-BP enrichment analysis, which revealed significant enrichment in monoatomic anion transport. Furthermore, several terms related to cell motility, including “cell migration” and “regulation of locomotion”, were also enriched. The shared DEPs included several established markers of the migratory machinery. Specifically, we identified the adhesion receptors ITGA5 and ITGAV, the actin-remodeling scaffold ABI1, the mTORC2 component RICTOR, and the junctional protein CTNND1. (Figure 5B,C, Supplementary Table S8). These findings indicate that the commonly regulated proteins by TAZ, Kaiso and HIF1A-AS3 prominently influence cellular migration programs.

Protein–protein interaction (PPI) analysis further showed that the shared DEPs form a significantly interconnected network (*p* value < 0.05) (Figure 5D), indicating functional relatedness. Major hubs within this network included EFTUD2 and NCBP1, core components of the mRNA processing machinery, linking the shared DEPs to regulation of gene expression in HSCs. GPI, a central glycolytic enzyme, was also present, pointing to a role in metabolic reprogramming. In addition, RRAGC and RRAGD, regulators of mTOR signaling, connected the network to a pathway critically involved in HSC activation.

Taken together, these data support a model in which TAZ, Kaiso, and HIF1A-AS3 converge on a shared downstream proteomic network that is involved in migration and integrates mRNA processing machinery with mTOR signaling components.

## 3. Discussion

The Hippo signaling pathway and its effectors YAP and TAZ are increasingly recognized as key regulators of HSC activation in liver fibrosis [15,19,20]. However, the mechanisms by which they orchestrate fibrogenic programs remain incompletely defined. Most studies have focused on their canonical partners, such as TEADs, leaving non-canonical interactors and downstream targets, including lncRNAs, largely unexplored [51,52]. In this study, we address this gap by identifying the transcription factor Kaiso as a novel, non-canonical TAZ interaction partner that exerts profibrotic effects on HSC activation. Furthermore, we identify HIF1A-AS3 as a downstream profibrotic lncRNA regulated by both TAZ and Kaiso.

Non-canonical YAP/TAZ interactors mapped using BioID revealed a broad and diverse network of novel interaction partners. This suggests that YAP/TAZ operate through multiple pathways beyond the canonical TEAD-mediated Hippo signaling. Our data also show that while YAP and TAZ share a core set of partners, each also engages a distinct subset of exclusive interactors, explaining how these paralogs mediate both overlapping and distinct functions, as reported in prior studies [53]. Interestingly, our results reveal a striking asymmetry wherein TAZ-exclusive partners outnumbered those of YAP by nearly fivefold in HSCs. This stands in direct contrast to hepatocytes, where YAP engages a more expansive interactome than TAZ [54]. Such divergence underscores the cell type-specific nature of YAP/TAZ interactions, suggesting that TAZ may support broader functions in HSCs, whereas YAP appears to play a more dominant role in hepatocytes.

Enrichment analysis indicates that this asymmetry in the YAP and TAZ interactomes in HSCs is functionally meaningful. The YAP-exclusive interactome, being relatively limited in size, was primarily focused on cytoskeletal remodeling. In contrast, the broader TAZ-exclusive interactome exhibited greater functional diversity, with strong enrichment in processes related to cell-cycle regulation, pointing to a specific role for TAZ in HSC proliferation. Additionally, we show that TAZ interactions were associated with other pathways central to HSC activation, including Wnt signaling, RNA metabolism, and cell migration. Collectively, these findings imply that TAZ orchestrates broader functions in HSCs.

Building on these results, we focused on the TAZ interactome and identified Kaiso as a particularly intriguing candidate, as it has never been investigated in liver fibrosis despite evidence linking it to the regulation of fibrotic pathways in other contexts [40,41,42,43,44,45]. Notably, a previous BioID study in lung cancer cells had listed Kaiso as a potential TAZ interactor, but that observation remained unvalidated [55]. Here, we confirm this interaction for the first time in the context of HSCs, revealing an unrecognized axis with potential relevance to liver fibrosis. This TAZ/Kaiso interaction expands the repertoire of Kaiso-interacting proteins beyond its well-characterized partners, including p120-catenin, δ-catenin, CTCF, TRIM28, and the NCoR complex [56,57,58,59,60].

In addition to its interaction with TAZ, our study demonstrates that the transcription factor Kaiso is also involved in the transcriptional activation of the lncRNA HIF1A-AS3. This lncRNA was identified through NGS analysis as a downstream target of YAP/TAZ signaling. Notably, to our knowledge, HIF1A-AS3 has only one previously identified transcriptional regulator, HIF1A, which induces its expression under hypoxic conditions [61]. These findings implicate Kaiso in hypoxia-related signaling, a particularly intriguing observation considering the role of hypoxia in HSC activation, which warrants further investigation [62].

Together, our findings point to a potential cooperative role for TAZ and Kaiso in the regulation of HIF1A-AS3. This scenario is reminiscent of the TAZ/TEAD model, in which TAZ lacks intrinsic transcriptional activity and therefore recruits TEAD to modulate downstream targets. Similarly, TAZ may depend on Kaiso to drive HIF1A-AS3 transcription, suggesting a potential TAZ/Kaiso/HIF1A-AS3 axis. Comparative analysis of our global proteomic results revealed a highly overlapping set of DEPs across TAZ, Kaiso, and HIF1A-AS3 knockdowns, further supporting the presence of a TAZ/Kaiso/HIF1A-AS3 regulatory axis. This inference is conceptually consistent with recent genome-wide proteomic studies, where similarity in proteomic signatures across distinct gene deletions has been used to infer functional convergence and define regulatory axes [63].

Our functional analyses indicate that Kaiso and HIF1A-AS3 act as positive regulators of HSC activation, mirroring the profibrotic effect previously described for TAZ [15]. Their impact was particularly pronounced on cell migration. Supporting these functional observations, our proteomics data revealed that common DEPs regulated by TAZ/Kaiso/HIF1A-AS3 include multiple migration-associated markers and are predominantly enriched in processes related to cell motility. Together, these findings provide a mechanistic explanation for why the strongest functional effects were observed in migration rather than in proliferation or colony formation. In addition, many of the shared DEPs were involved in monoatomic anion transport, a process that has recently been linked to HSC activation and α-SMA expression [64]. Network analysis further demonstrated that the shared DEPs form a significantly interconnected PPI module, indicating coordinated functional response extending beyond migration to include post-transcriptional gene regulation, metabolic reprogramming, and mTOR signaling. Taken together, these findings suggest that Kaiso and HIF1A-AS3 could be components of the Hippo signaling molecular machinery that regulate HSC activation.

Notably, the profibrotic effects of Kaiso identified in HSCs in this study are consistent with its previously reported profibrotic role in the lung [45], and provide a potential mechanistic link to its involvement in the transition from MASLD to HCC [47]. These findings also align with extensive evidence connecting Kaiso to enhanced proliferation across multiple cancer types, including HCC [43,46,65,66]. Furthermore, our newly recognized profibrotic role for HIF1A-AS3 in HSCs resembles that of other hypoxia-responsive genes within the same locus, including HIF1A and HIF1A-AS1 [67,68,69]. Our findings are also consistent with evidence that HIF1A-AS3 activates fibrogenic programs in mesenchymal stromal cells [70], and align with its reported activity on cell behavior in lung cancer, where it promotes proliferation and invasion [71].

Overall, our findings provide a foundation for defining the roles of Kaiso and HIF1A-AS3 in HSC activation and broaden the current understanding of the molecular mechanisms underlying hepatic fibrogenesis. Building on these findings, future studies could further elucidate the functional and mechanistic significance of the TAZ/Kaiso/HIF1A-AS3 axis. Gain-of-function experiments would provide complementary evidence by determining whether overexpression of Kaiso or HIF1A-AS3 is sufficient to enhance HSC activation and further establish their roles in HSC fibrogenic activity. Moreover, although our findings demonstrate an interaction between TAZ and Kaiso and reveal similar functional outputs in HSCs, the mechanistic significance of this partnership remains to be fully defined. Future studies should therefore determine how the TAZ/Kaiso interaction influences their transcriptional activity and contributes to the regulation of fibrogenic genes. Furthermore, given the established association of Kaiso with tumorigenic processes and the potential link between liver fibrosis and hepatocarcinogenesis, investigating the role of Kaiso and the TAZ/Kaiso/HIF1A-AS3 axis in HCC and in the progression from fibrosis to tumor development could provide further insight into the broader significance of this pathway.

A limitation of the present study is that all experiments were conducted using a single HSC cell line, LX-2. Although LX-2 provides a well-established model for mechanistic studies of hepatic fibrogenesis, validation of our findings in additional HSC models, particularly primary human HSCs, would strengthen the generalizability of our findings and provide further support for the potential relationship between TAZ, Kaiso, and HIF1A-AS3. Moreover, investigating the expression and function of these mediators in quiescent HSCs and during the transition toward an activated phenotype would provide further insight into their role in HSC biology. Taken together, these considerations highlight the need for future studies using primary HSCs and in vivo models to further define the individual contribution of TAZ, Kaiso, and HIF1A-AS3, validate their proposed regulatory axis, and determine whether targeting these mediators individually or as a network can effectively attenuate liver fibrogenesis.

In conclusion, this study investigated mediators of YAP/TAZ-driven fibrogenic activity in HSCs, focusing on novel interaction partners and downstream lncRNAs. We identified Kaiso as a TAZ- interactor with profibrotic activity and showed that the lncRNA HIF1A-AS3 is regulated by both TAZ and Kaiso and contributes to HSC activation. We also show that TAZ, Kaiso, and HIF1A-AS3 share a highly correlated proteomic signature. Together, these findings describe a potential TAZ/Kaiso/HIF1A-AS3 axis that may coordinate fibrogenic programs in HSCs and warrant further investigation as a possible therapeutic target in liver fibrosis.

## 4. Materials and Methods

### 4.1. Cell Culture and Transfection

The human hepatic stellate cell line LX-2 (SCC064, Millipore, Darmstadt, Germany) was used in this study. LX-2 was selected because it is the only commercially available human HSC line currently accessible through global vendors, whereas primary HSCs are limited by tissue availability, donor variability, and low cell yield, and LX-1 cells are not commercially available. In addition, LX-2 offers a key technical advantage for our extensive genetic manipulation experiments, as LX-1 cells and primary human HSCs have been reported to exhibit very low transfection efficiency (<1%) compared with LX-2 cells [38]. Cells were maintained for passaging and transfection in Dulbecco’s Modified Eagle Medium (DMEM; BioWest, Nuaillé, France) supplemented with 2% fetal bovine serum (FBS; Gibco, Grand Island, NY, USA) and 1% penicillin-streptomycin (Lonza, Basel, Switzerland). Cells were cultured at 37 °C in a humidified incubator containing 5% CO_2_. No exogenous pro-fibrotic stimulus was added. For transfection, cells were seeded 24 h prior to treatment in 6- or 12-well plates to reach approximately 70% confluency, depending on the downstream assay. Transfections were performed with Lipofectamine 3000 (Thermo Fisher Scientific, Waltham, MA, USA) using either 40 nM target-specific or corresponding negative control (NC) oligonucleotides for individual knockdowns, or 30 nM of each oligonucleotide for combined knockdowns (60 nM total), with a matching 60 nM negative control. All transfections were carried out according to the manufacturer’s instructions. Cells were harvested 24 or 48 h post-transfection for downstream analyses. Details of all oligonucleotides, including catalogue numbers and sequences, are provided in Supplementary Table S1.

### 4.2. Biotin Identification, Proximity Labeling and Proteomic Analysis

#### 4.2.1. Stable Transfection with BirA-YAP and BirA-TAZ

For Biotin Identification (BioID) assays, we generated lentiviral constructs as previously described [54] to establish LX-2 cells stably expressing BirA-Flag-tagged YAP or TAZ. Briefly, human YAP and TAZ cDNAs (gifts from Dr. Xiaolong Yang and Dr. Xaralabos Varelas, respectively) were cloned into a pDONR entry vector and subsequently transferred by Gateway^®^ LR recombination into the pTRIPz lentiviral destination vector to generate N-terminally tagged BirA-Flag-YAP and BirA-Flag-TAZ constructs. All constructs were verified by sequencing. Lentiviral particles were produced in HEK293T cells and used to transduce LX-2 cells in the presence of polybrene (8 µg/mL). Stably transduced cells were selected with puromycin (2 µg/mL).

#### 4.2.2. BioID Pulldown

BioID pulldown was performed as described previously [54], with adaptations for LX-2 cells. Briefly, cells expressing BirA fusion proteins were seeded on 15 cm dishes (2 × 10^6^ cells/dish). Fusion protein expression was induced with 1 µg/mL doxycycline for 24 h, followed by supplementation with 50 µM biotin (Sigma-Aldrich, St. Louis, MO, USA) for an additional 24 h. Cells were lysed in BioID lysis buffer (50 mM Tris pH 7.5, 200 mM NaCl, 0.1% SDS, 1% Triton X-100, 1 mM EDTA, 0.25% sodium deoxycholate), sonicated, and cleared by centrifugation. Biotinylated proteins were enriched using Pierce™ Streptavidin Magnetic Beads (Thermo Fisher Scientific), subjected to sequential high-stringency washes, and eluted with biotin and Laemmli buffer. Eluted proteins were analyzed by LC/MS.

#### 4.2.3. Proteomic Sample Preparation and LC/MS Analysis

Upon SDS-PAGE, Coomassie-stained bands were manually excised from the gel. The in-gel digestion was performed as described earlier [72,73]. Samples were suspended in 0.1% TFA and analyzed using an Ultimate 3000 liquid chromatography system coupled to an Orbitrap QE HF (Thermo Fisher Scientific) as described before [73]. Briefly, peptides were separated in a 60 min linear gradient starting from 3% B and increasing to 23% B over 50 min and to 38% B over 10 min, followed by washout with 95% B. The mass spectrometer was operated in data-dependent acquisition mode, automatically switching between MS and MS2. MS spectra (m/z 400–1600) were acquired in the Orbitrap at 60,000 (m/z 400) resolution, and MS2 spectra were generated for up to 15 precursors with a normalized collision energy of 27 and an isolation width of 1.4 m/z. The MS/MS spectra were searched against the UniProt H. sapiens (UP000005640, downloaded June 2020) and a customized contaminant database using MaxQuant (version 1.6.12.0) and the Andromeda search engine [74,75]. Trypsin was specified as an enzyme, allowing up to 2 missed cleavages. The following variable modifications were allowed: Oxidation (M), Deamidation (N, Q), Acetylation (N-terminus), whereas Carbamidomethylation (C) was set as a fixed modification. The maximum false discovery rate for proteins and peptides was 0.01 with a minimum peptide length of seven amino acids. Quantitative normalized ratios were calculated by MaxQuant and subsequently analyzed with Perseus (version 1.16.15.02) [76] and STRING (accessed August 2026) [77]. Interactome overlaps were visualized using Venn diagrams generated with InteractiVenn (accessed September, 2025) [78], and transcription factors were filtered using the AnimalTFDB database v4.0 (filtering for *Homo sapiens)* [79]. To infer Kaiso transcription factor activity across HSC states, we used a recently published single-cell HSC atlas [48]. BioID experiments were performed in four independent biological replicates.

### 4.3. Proximity Ligation Assay

PLA assays were performed using the Duolink^®^ In Situ PLA^®^ system (Merck, Darmstadt, Germany). LX-2 cells were seeded at a density of 2 × 10^5^ cells on 12 mm glass coverslips in 6-well plates. The following day, cells were washed with PBS, fixed with 4% paraformaldehyde, washed again, permeabilized with 0.2% Triton X-100, and blocked with BSA blocking solution for 30 min at room temperature in a humidified chamber. Cells were then incubated overnight at 4 °C with rabbit anti-Kaiso and/or mouse anti-TAZ primary antibodies (antibody details, dilutions, and catalog numbers are provided in Supplementary Table S2).

Following primary antibody incubation, coverslips were washed twice and incubated for 1 h at 37 °C with Duolink^®^ In Situ PLA^®^ Probe Anti-Rabbit PLUS and Duolink^®^ In Situ PLA^®^ Probe Anti-Mouse MINUS. After washing, coverslips were incubated with Duolink^®^ In Situ PLA^®^ ligation reagents, and the ligation reaction was carried out for 30 min at 37 °C. Subsequently, polymerase and amplification solution were applied, and coverslips were incubated for 100 min at 37 °C in a humidified chamber. Following amplification, coverslips were washed, air-dried, and mounted using Fluoromount-G containing DAPI supplemented with Alexa Fluor 488-conjugated phalloidin (1:2000). Slides were allowed to cure overnight at 4 °C before storage at −20 °C until imaging. PLA signals were visualized using a BZ-X810 fluorescence microscope (Keyence, Osaka, Japan) equipped with a 100 × objective. All images were acquired using identical exposure and image acquisition settings across experimental groups and processed using BZ-X800 Analyzer software (version 1.3.0; Keyence, Osaka, Japan).

### 4.4. RNA Extraction and qRT-PCR

For RNA extraction, total RNA was extracted using the miRNeasy Kit (Qiagen, Hilden, Germany), according to the manufacturer’s instructions. RNA quantity and purity were assessed spectrophotometrically, and 1 *μ*g was reverse-transcribed into cDNA with the High-Capacity cDNA Reverse Transcription Kit (Applied Biosystems, Waltham, MA, USA). Quantitative real-time PCR (qRT-PCR) was performed using RT^2^ SYBR Green qPCR Mastermix (Qiagen) on a QuantStudio instrument (Applied Biosystems). Relative expression levels were calculated using the comparative CT method (2^−ΔΔCT^), with GAPDH serving as the housekeeping gene for normalization. Primer sequences are provided in Supplementary Table S3.

### 4.5. Protein Extraction and Western Blot

For protein extraction, cells were lysed with RIPA lysis buffer (Serva, Heidelberg, Germany) supplemented with a 1 × protease and phosphatase inhibitor cocktail (Thermo Fisher Scientific). Lysates were incubated on a thermoshaker for 30 min at 4 °C, followed by centrifugation to pellet cellular debris. The resulting supernatants were collected, and protein concentrations were determined using a Modified Lowry Protein Assay Kit (Thermo Fisher Scientific). Equal amounts of protein were separated by SDS-PAGE and transferred onto nitrocellulose membranes. Membranes were blocked for 30 min at room temperature with 3% BSA in TBST, incubated overnight at 4 °C with primary antibodies, and, after washing, incubated for 1 h at room temperature with the appropriate HRP-conjugated secondary antibodies. Protein bands were visualized using an ImageQuant LAS 500 system (GE Healthcare, Uppsala, Sweden) and quantified with ImageQuant software (version 8.2.0). For quantification, band intensities were measured densitometrically, and target protein levels were normalized to their corresponding loading control. Normalized values were then expressed as fold change relative to the NC. Details of all antibodies used are provided in Supplementary Table S2.

### 4.6. Immunofluorescence

For immunofluorescence (IF) staining, cells were seeded onto sterile round glass coverslips and cultured under standard conditions. They were fixed with 4% paraformaldehyde (Piochem, Giza, Egypt) for 15 min at room temperature and subsequently permeabilized with 0.2% Triton X-100 (BioBasic, Markham, ON, Canada) for 10 min. To block nonspecific antibody binding, coverslips were incubated with 1% BSA (BioWest) for 1 h at room temperature. Cells were then incubated overnight at 4 °C with a primary antibody against α-SMA. After that, they were washed and subsequently incubated with the appropriate Alexa Fluor-conjugated secondary antibody for 1 h at 37 °C. Details of antibodies are provided in Supplementary Table S2.

Coverslips were mounted using ProLong™ Antifade Mountant with DAPI (Cell Signaling Technology, Danvers, MA, USA). Images were acquired at 40× magnification using a fluorescence microscope (DMC4500; Leica Microsystems, Wetzlar, Germany), equipped with LAS X software (version 3.7). Immunofluorescence experiments were performed in two independent biological replicates, with 5 random fields analyzed per condition. Nuclei were identified and counted using QuPath (version 0.5.1). α-SMA fluorescence intensity was quantified using an ImageJ (version 1.54p) plugin within QuPath following background subtraction. Mean fluorescence intensity (MFI) was calculated by dividing the total integrated α-SMA signal by the number of nuclei per image. MFI values were normalized to their respective negative controls. All images were acquired and processed using identical settings across experimental groups.

### 4.7. Chromatin Immunoprecipitation (ChIP)

#### 4.7.1. ChIP-Seq Analysis

To identify lncRNA genes bound by Kaiso, publicly available ChIP-seq data (GEO accession: GSE169918) aligned to the GRCh38 human reference genome were analyzed. High-confidence binding events were defined using optimal irreproducible discovery rate (IDR)–thresholded peaks. Peak annotation was performed in R using the ChIPseeker package (version 1.44.0), with promoter regions defined as ±2 kb from transcription start sites (TSS) based on the TxDb.Hsapiens.UCSC.hg38.knownGene annotation database. Gene biotype information was retrieved from Ensembl using the biomaRt package to distinguish lncRNAs from protein-coding genes.

#### 4.7.2. ChIP-qPCR

ChIP-qPCR was performed as previously described [80]. To validate Kaiso binding, peaks were visualized using the NCBI Genome Data Viewer within a ±2 kb region surrounding the transcription start site (TSS). Primer pairs were designed to amplify DNA sequences corresponding to the identified peak region. A primer pair targeting a random genomic region lacking Kaiso binding was used as a negative control. Following immunoprecipitation, DNA was purified using the QIAquick PCR Purification Kit and analyzed by qPCR using input DNA for normalization. Enrichment was expressed as a percentage of input (%Input). Antibody details are provided in Supplementary Table S2, and primer sequences are listed in Supplementary Table S3.

### 4.8. Next-Generation Sequencing (NGS)

To identify lncRNAs regulated by YAP and TAZ, LX-2 cells were transfected with siRNA combinations targeting YAP/TAZ (40 nM, si-YAP/TAZ). Total RNA was extracted 24 h post-transfection using the NucleoSpin RNA II kit (Macherey-Nagel, Düren, Germany). Samples with RNA integrity numbers (RIN) greater than 7 were selected for sequencing (BGI, Hong Kong, China). Data analysis was performed in R/Bioconductor using the systemPipeR framework. Raw read quality was assessed with FastQC, and adapters were trimmed with Trim Galore. Processed reads were quantified using kallisto quant (version 0.4.6) against a human transcriptome reference based on GRCh38.p13. Differential expression analysis was carried out with the limma package on log2-counts per million (logCPM)-transformed data, with statistical significance defined as a false discovery rate (FDR) < 0.05.

### 4.9. BrdU Cell Proliferation Assay

To assess cell proliferation, transfected cells were harvested, counted, and reseeded at a density of 6000 cells per well in 96-well plates. The following day, cell proliferation was quantified using a BrdU ELISA kit (Roche, Basel, Switzerland). Cells were incubated with BrdU (5-bromo-2′-deoxyuridine) labeling solution for 4 h at 37 °C under standard culture conditions. After labeling, the assay was completed according to the manufacturer’s instructions. Absorbance was measured at 370 nm using a microplate reader, and proliferation was expressed as the percentage of treated cells relative to negative control cells.

### 4.10. Colony Formation

Transfected cells were harvested, counted, and reseeded at low density in 6-well plates 24 h post-transfection. Cells were then incubated for 15 days to allow colony formation. Once colonies had developed, they were stained with 0.05% crystal violet (Rankem, Delhi, India) for 1 h at room temperature, after which they were counted and analyzed. The experiment was performed in two independent biological replicates. The relative colony number was calculated by normalizing to the corresponding negative control group.

### 4.11. Transwell Migration Assay

Cells were harvested 24 h post-transfection, counted, and reseeded at a density of 5 × 10^4^ cells in serum-free medium into the upper chamber of a 24-well Transwell insert (8 μm pore size; Greiner Bio-One, Frickenhausen, Germany). DMEM supplemented with 10% FBS as a chemoattractant was added to the lower chamber. Cells were then incubated at 37 °C for 24 h to allow migration. Following incubation, the medium was aspirated, and non-migrated cells on the upper surface of the membrane were carefully removed using a cotton swab. Migrated cells on the lower surface were fixed with 4% paraformaldehyde and permeabilized with 0.02% Triton X-100. Cell nuclei were stained using ProLong™ Gold Antifade Mountant (Thermo Fisher Scientific) with DAPI. Images were acquired using a fluorescence microscope (Leica Microsystems, DMC4500). For quantification, six random fields per insert were captured at 20× magnification. The number of migrated cells was counted using QuPath software (v0.5.1). The migration rate was calculated as a percentage normalized to the NC group.

### 4.12. Shotgun Proteomics Analysis

#### 4.12.1. Preparation of Protein Lysates

Cells were rinsed with PBS and pelleted by centrifugation at 10,000 rpm. The pellets were lysed in 100 µL of lysis buffer (8 M urea, 500 mM Tris-HCl, pH 8.5) supplemented with Complete Ultra protease inhibitor cocktail (Roche, Mannheim, Germany). Samples were incubated at 37 °C for 1 h with intermittent vortexing, followed by centrifugation at 12,000 rpm for 20 min. Protein concentration in the supernatant was determined using the BCA assay (Pierce, Rockford, IL, USA) at 562 nm [81,82].

#### 4.12.2. In-Solution Tryptic Digestion

For each sample, 30 µg of protein was reduced with 200 mM dithiothreitol (DTT) in 8 M urea buffer for 30 min, then alkylated with 10 mM iodoacetamide for 30 min in the dark. Samples were diluted to 2 M urea using 100 mM Tris-HCl (pH 8.5) prior to digestion. Sequencing-grade modified porcine trypsin (Sigma, St. Louis, MO, USA) was added at a 30:1 protein-to-enzyme ratio (*w*/*w*), and digestion was carried out overnight at 37 °C with shaking at 600 rpm. The reaction was quenched by acidification to pH 2.0 with formic acid. Peptides were desalted using StageTips as described previously and quantified by BCA assay. Final peptide solutions were adjusted to 1 µg/10 µL for LC-MS/MS analysis [83].

#### 4.12.3. LC–MS/MS Analysis

Peptides (100 ng) were analyzed using an EASY-nanoLC 1200 system coupled to an Orbitrap Fusion Lumos Tribrid mass spectrometer (Thermo Fisher Scientific) via a NanoFlex source. Peptides were loaded onto a Thermo Scientific™ Acclaim™ PepMap™ 100 trap column (75 µm × 2 cm, 3 µm C18) and separated on an analytical column of the same chemistry (75 µm × 25 cm, 2 µm C18). Elution was performed with a 60 min gradient of solvent B (80% acetonitrile, 0.1% formic acid) at 250 nL/min as follows: 5–30% (0–31 min), 30–40% (31–41 min), 40–80% (41–51 min), held for 4 min, then ramped to 100% for 5 min. Solvent A was 0.1% formic acid in water. Each sample was run in technical replicates to ensure reproducibility [82].

The mass spectrometer operated in data-dependent acquisition (DDA) mode with 3 s duty cycles. MS1 scans were acquired at 120,000 resolution over 400–1800 m/z with standard AGC and automatic injection time. Monoisotopic precursor selection (MIPS) was enabled with an intensity threshold of 5 × 10^3^, selecting charge states 2–7. Dynamic exclusion was set to 30 s with a 10 ppm mass tolerance. MS2 spectra were acquired in the ion trap following HCD fragmentation at 30% collision energy using a 1.5 m/z isolation window [84].

#### 4.12.4. Data Processing and Protein Identification

Raw data were processed using Proteome Discoverer (v2.4.0.305; Thermo Fisher Scientific). Spectra were searched against the UniProt Homo sapiens reference proteome (2022 release; 225,732 entries) using the Sequest HT algorithm. Search parameters allowed for fully and semi-tryptic peptides with up to two missed cleavages and a minimum peptide length of 6 amino acids. Mass tolerances were set to 20 ppm for precursors and 0.5 Da for fragments. Carbamidomethylation of cysteine (+57.021 Da) was set as a fixed modification, while oxidation of methionine (+15.995 Da), N-terminal acetylation (+42.010 Da), and conversion of carbamidomethylated cysteine to pyrrolidone (−17.030 Da) were set as variable modifications [84].

Label-free quantification was performed using the Minora Feature Detector. Peptide and protein false discovery rates (FDR) were controlled at 1% using a decoy database strategy. Quantitative values were normalized using Total Peptide Amount and scaled across all samples. Differential expression was calculated as treatment-to-control ratios (log2FC ≥ 1, *p* ≤ 0.05). Differential protein abundance statistics were obtained directly from Proteome Discoverer. Significant proteins (FDR < 0.05, |log2FC| ≥ 1) were identified for three treatment-versus-control contrasts. Protein identifiers were mapped from UniProt accessions to gene symbols, isoform information was standardized, and duplicate mappings were collapsed at the gene level to avoid redundancy. Overlaps between each of the three datasets were visualized using Venn diagrams generated with InteractiVenn. Overlap gene sets were computed across contrasts without considering directionality in order to identify genes consistently altered across multiple experimental conditions. Over-representation analysis was then performed on these overlap sets using Gene Ontology (GO) biological process annotations. Multiple testing correction was applied using Benjamini–Hochberg. Significantly enriched GO terms (adjusted *p* < 0.05) were visualized as bar plots in addition to a gene-concept network, where nodes represent genes or GO terms and edges indicate gene–term associations. Node size reflects the number of genes contributing to each GO term. Bar plots display the top enriched GO terms ranked by adjusted *p*-value, while network representations illustrate gene–term connectivity. PPI analysis was performed for the shared differentially expressed proteins using the STRING database [77,82]. Interactions were retrieved based on co-expression and experimentally determined evidence channels. The resulting interaction network was imported into Cytoscape (version 3.10.3) for visualization [83], where edge colors represent interaction evidence type and edge thickness reflects interaction confidence, as defined by STRING.

### 4.13. Statistical Analysis

Experiments were performed in three independent biological replicates unless stated otherwise. Data are presented as mean ± standard error of the mean (SEM). Statistical significance between two groups was assessed using an unpaired Student’s *t*-test after confirming normality. A *p*-value < 0.05 was considered significant, denoted as follows: * *p* < 0.05, ** *p* < 0.01, *** *p* < 0.001. All statistical analyses, including Spearman correlations for protein groups, were performed using GraphPad Prism (version 8.0.2).

## Data Availability

The NGS dataset generated during this study has been deposited in the NCBI Gene Expression Omnibus (GEO) database under accession number GSE345280. The mass spectrometry proteomics datasets have been deposited to the ProteomeXchange Consortium via the PRIDE [85] partner repository with dataset identifiers PXD074737 (BioID proteomics) and PXD081135 (whole-proteome mass spectrometry following TAZ, Kaiso, and HIF1A-AS3 knockdown).

## Supplementary Materials

The following supporting information can be downloaded at: https://www.mdpi.com/article/10.3390/ijms27177847/s1.

## Abbreviations

The following abbreviations are used in this manuscript:

α-SMA	Alpha-smooth muscle actin
BioID	Proximity-dependent Biotin Identification
ChIP	Chromatin immunoprecipitation
DEPs	Differentially expressed proteins
FDR	False discovery rate
GO-BP	Gene Ontology Biological Process
HIF1A-AS3	Hypoxia-inducible factor 1 alpha antisense RNA 3
HSC	Hepatic stellate cell
IF	Immunofluorescence
lncRNAs	Long non-coding RNAs
MFI	Mean fluorescence intensity
NC	Negative control
NGS	Next-generation sequencing
PPI	Protein–protein interaction
qRT-PCR	Quantitative real-time polymerase chain reaction
SEM	Standard error of the mean
TAZ	Transcriptional coactivator with PDZ-binding motif
TEAD	TEA domain transcription factor
TSS	Transcription start site
YAP	Yes-associated protein
