# Supplementary material for: Kaiso Is a Novel TAZ-Interaction Partner That Promotes Hepatic Stellate Cell Activation and Regulates the Downstream lncRNA HIF1A-AS3
Source: Int J Mol Sci. 2026 Sep 2;27(17):7847. doi: 10.3390/ijms27177847 (PMC13566411; doi:10.3390/ijms27177847)
Supplement: Supplementary file 1 [file ijms-27-07847-s001.zip › Supplementary_data_IJMS.pdf]

Supplementary Figures

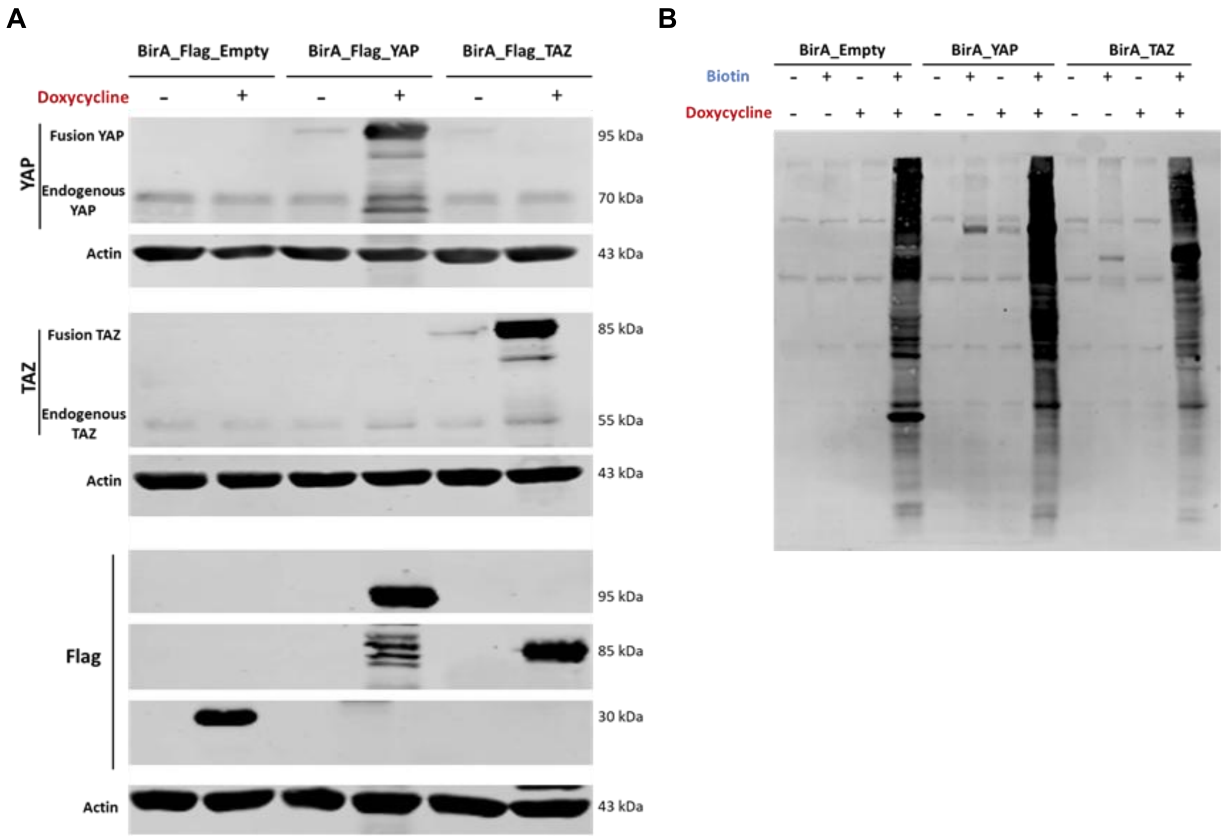

Supplementary Figure S1. Validation of BioID for YAP and TAZ in LX-2 Cells.

LX-2 cells were stably transfected with doxycycline-inducible BirA-Flag fusion constructs expressing either empty vector, YAP, or TAZ. (A) Immunoblot analysis of YAP, TAZ, and Flag expression in transfected cells after doxycycline treatment, confirming overexpression of the fusion proteins. Actin served as a loading control. (B) Streptavidin blot detecting biotinylated proteins in LX-2 cells expressing BirA-Flag constructs under the indicated conditions (biotin only, doxycycline only, or both), confirming successful biotinylation.

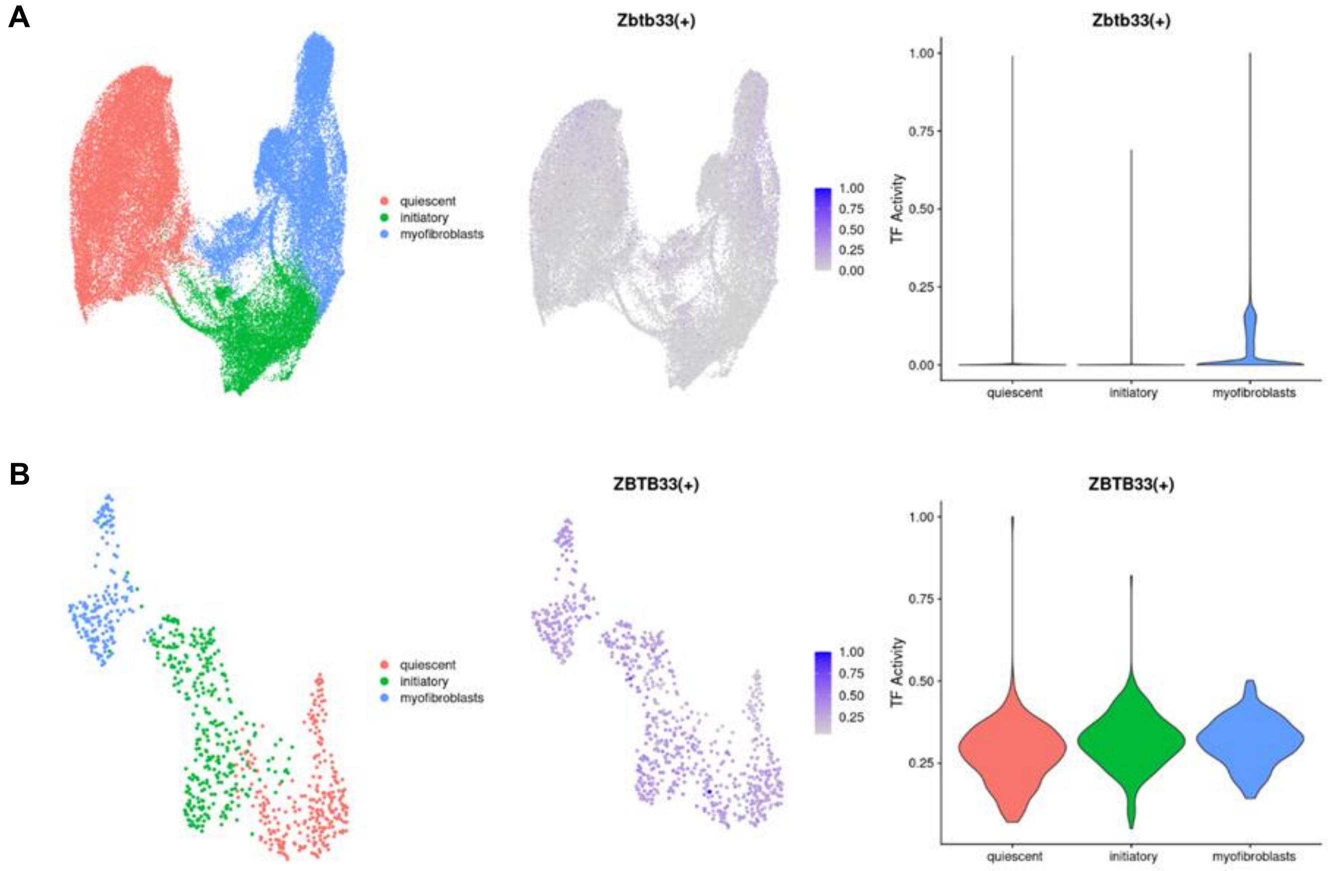

Supplementary Figure S2. Kaiso transcription factor activity in HSCs.

Kaiso (ZBTB33) transcription factor activity was obtained using the HSC Atlas Shiny App in (A) mouse datasets and (B) human datasets. All panels were generated directly from the atlas interface. (Left) Uniform Manifold Approximation and Projection (UMAP) identifying three HSC states: quiescent, initiatory, and myofibroblast. (Middle) Feature plot showing Kaiso transcription factor activity intensity overlaid onto the UMAP projection. (Right) Violin plots showing relative Kaiso activity across the three HSC states.

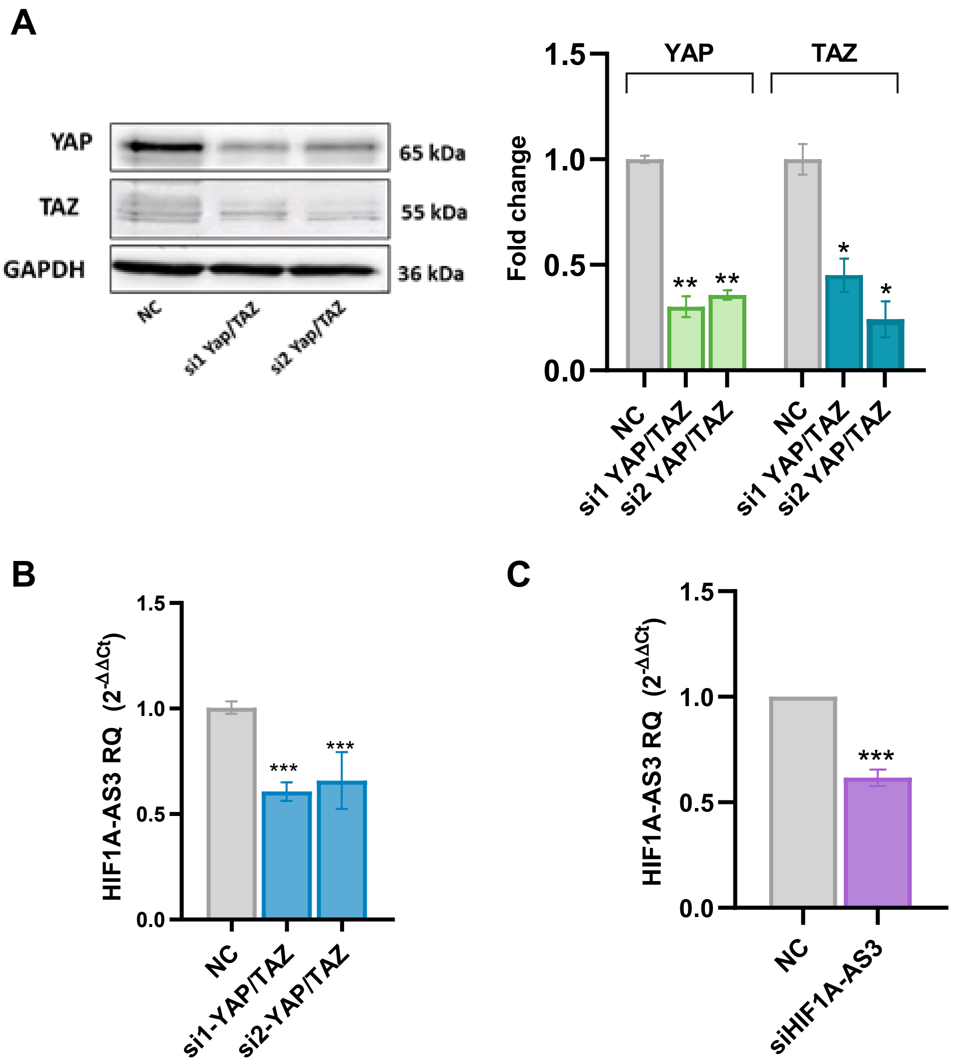

Supplementary Figure S3. Validation of knockdown efficiency and HIF1A-AS3 expression following YAP/TAZ silencing.

(A) Representative Western blots and corresponding quantification confirming knockdown of YAP and TAZ in siYAP/TAZ-transfected cells. GAPDH served as the loading control. (B) qRT-PCR analysis of HIF1A-AS3 expression following YAP/TAZ knockdown. (C) qRT-PCR analysis confirming efficient knockdown of HIF1A-AS3 in siHIF1A-AS3-transfected cells compared with the NC group.
